# Vision in the Vertical Axis: How Important Are Visual Cues in Foraging and Navigation?

**DOI:** 10.3390/vision7020044

**Published:** 2023-06-06

**Authors:** Jessica L. Campbell, Theresa Burt de Perera

**Affiliations:** Animal Behaviour Research Group, Department of Zoology, University of Oxford, South Parks Road, Oxford OX1 3PS, UK

**Keywords:** navigation, fish, three dimensions, cue conflict, vision

## Abstract

In both terrestrial and aquatic environments, a large number of animal behaviors rely on visual cues, with vision acting as the dominant sense for many fish. However, many other streams of information are available, and multiple cues may be incorporated simultaneously. Being free from the constraints of many of their terrestrial counterparts, fish have an expanded range of possible movements typified by a volume rather than an area. Cues such as hydrostatic pressure, which relates to navigation in a vertical plane, may provide more salient and reliable information to fish as they are not affected by poor light conditions or turbidity. Here, we tested banded tetra fish (*Astyanax fasciatus*) in a simple foraging task in order to determine whether visual cues would be prioritized over other salient information, most notably hydrostatic pressure gradients. We found that in both vertical and horizontal arrays there was no evidence for fish favoring one set of cues over the other, with subjects making choices at random once cues were placed into conflict. Visual cues remained as important in the vertical axis as they were in the horizontal axis.

## 1. Introduction

A large number of known animal behaviors, from foraging to courtship and navigation, rely on visual cues. Reliance on visual cues is in fact so widespread that it is not an exaggeration to assert that vision is central to biology [1]. This is no less true in aquatic environments than it is on land, where many fish have evolved complex visual systems including tri-, tetra-, and even pentachromacy and sensitivity to polarized light [2,3]. Such highly adapted systems, together with a wide range of bodily coloration and patterns, enable vision to act as the dominant sense of many fishes [4]. Visual cues are not, however, the only ones available; many animals have access to several sensory modalities and can use multiple cues in conjunction [5]. The parallel use of multiple systems does, however, come with an intensified information processing/decision-making load, and nowhere is this truer than in fish who, free from the two-dimensional constraints of many of their terrestrial counterparts, have an expanded range of possible movements typified by a volume rather than an area. Accordingly, they provide an excellent system in which to explore the relative importance of visual cues when alternative cues are available.

In addition to using visual landmarks [6,7,8], fish are able to access many other possible senses or sensory systems to aid in navigation when foraging or looking for shelter. These include but are not limited to olfaction [9], audition [10], the lateral line [11], electrolocation [12], and proprioception. As recently elucidated, fish are also able to make use of hydrostatic pressure gradients in their environment [13], possibly using the swim bladder [14]. Multiple senses may be combined in order to create a map integrating multisensory information, or they can be used redundantly when one set of information is lost [15]. Which senses (and cues) are used in any given setting may be influenced by many factors including the ecology of the species, habitat stability, and the environmental conditions at the time. It may be hypothesized that animals are, in general, more likely to place importance on cues which are more reliable or salient in their usual setting, favoring the corresponding sensory system. In this respect, hydrostatic pressure is particularly interesting to compare with visual cues. While vision is undoubtedly important, visual cues also become unreliable during poor light conditions or when water turbidity increases. In contrast, hydrostatic pressure is reliable and stable over time and space [14,16] and, as demonstrated by Davis et al. [13], can act not only as a gradient, giving information about movement in the vertical axis, but also as a distance-based cue that can allow fish precise localization of their vertical position. Accordingly, the question of whether visual or hydrostatic pressure cues are preferred by fish is pertinent. One way to address this question is by placing these cues into conflict.

There are multiple ways of placing cues into conflict. One such method is by using a transposition task, as has been used in bees and hummingbirds [17,18]. Transposition requires animals to be capable of discriminating between two stimuli presented in a predictable relationship. Animals are trained to visit one of two stimuli, learning a rule such as ‘chose the upper landmark’. All landmarks are then shifted in their absolute position (in this case, upwards), but the relative position is maintained. To show transposition, the animal would continue to rely on the previously learnt rule and would therefore visit the upper landmark (which is now in a novel location). Such behavior would necessitate that subjects were paying attention to the position of stimuli in relation to each other (rather than their absolute position in space) and therefore would be relying heavily on visual cues. It cannot be ruled out that, in fish, landmarks may also be sensed using the lateral line or other mechanosensory systems; however, in clear water, it is expected that vision would dominate. In contrast, any individual selecting the previously reinforced landmark would have access to other cues which signal an absolute position in space. In an aquatic setting, in the vertical axis, this would notably include hydrostatic pressure, which may be a more robust and reliable cue than vision under certain circumstances.

Previous work has shown mixed results when cues are placed into conflict. In experiments with banded tetra fish (*Astyanax fasciatus*), landmarks override egocentric cues (directional cues based on the orientation of their body) when the two are put into conflict [19], despite both sets of cues having previously been equally reliable. Odling-Smee [20] similarly found that sticklebacks from pond habitats favored landmarks, though those from river habitats were more likely to use egocentric cues. In contrast, damselfish strongly favored egocentric rather than visual cues in a range of turbidities, surprisingly including in clear water [21]. Regarding differences between axes, both banded tetras and bronze Corydoras (*C. aeneus*) have been shown to prefer vertical information and disregard visual landmarks and horizontal information, respectively, in Y-maze tasks [22,23]. As such, there still remains uncertainty about cue use in different taxa and contexts, with few experiments so far conducted looking at free-swimming fish.

Here, we used banded tetra fish (*Astyanax fasciatus*) in a simple foraging task in order to test whether visual cues would be prioritized over other salient information including hydrostatic pressure gradients. Specifically, we wished to determine whether visual cues were equally important in the horizontal and vertical axes. Work by Holbrook and de Perera [24] suggests that banded tetra fish strongly favor vertical cues over horizontal cues, perhaps making use of hydrostatic pressure gradients. By making use of pressure gradients, fish may therefore be capable of learning the absolute height of a rewarded landmark, as shown by Davis et al. [13]. Since hydrostatic pressure cues are unavailable in the horizontal axis, a switch to using relational visual based cues (e.g., learning a rule such as ‘always swim to the left-hand landmark’) may be expected. This leads to the hypothesis that fish will behave differently in horizontal and vertical tests, using visual cues (visiting the novel landmark) in the horizontal axis and alternative positional cues (visiting the previously reinforced landmark) in the vertical axis.

## 2. Materials and Methods

### 2.1. Subjects

The subjects were 16 captive-bred banded tetra fish (*Astyanax fasciatus*) from a population originally collected from Texas, U.S.A. All the fish were experimentally naïve. During the experimental training, two fish were removed from further trials due to the failure to learn the task and poor health. The data presented in this paper are therefore based on the 14 remaining fish.

Fish were housed in 0.45 m × 0.30 m × 0.30 m aquaria divided into four compartments (or 0.60 m × 0.30 m × 0.30 m aquaria divided into five compartments). One compartment contained a biological filter and the other three/four compartments contained one fish each. Each fish-containing compartment additionally contained an air stone connected to an air pump and java moss to act as enrichment and a refuge. Compartments were separated using clear, perforated Penn-Plax dividers to allow visual and chemical interaction between individuals in adjacent compartments. Aquaria were situated in a laboratory kept at 24–25 °C by a constant-temperature air-conditioning apparatus and were lit overhead by fluorescent lights on a 12:12 diurnal cycle. During the experimental phase, subjects received food only within the testing tank.

This study was approved by the Local Ethics Review Committee in the Department of Zoology, Oxford, and did not require a Home Office License.

### 2.2. Experimental Setup

**Figure 1:**
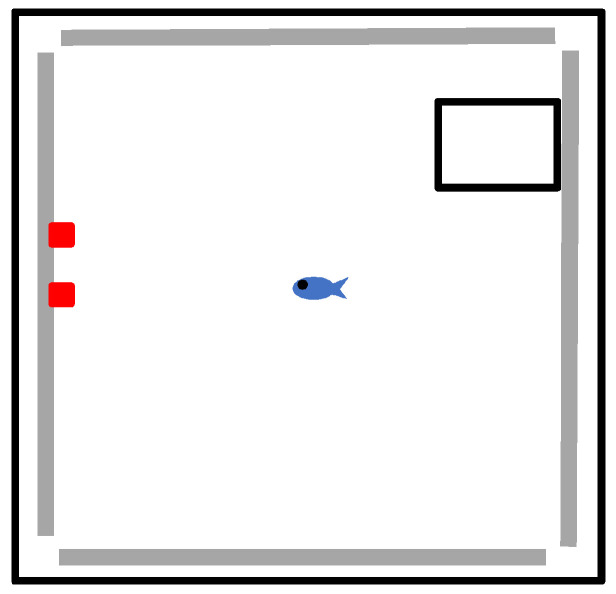
Bird’s-eye view of the experimental setup showing the testing arena with the holding box to the right and red Lego landmarks on the left-hand baseplate.

The testing arena consisted of a large white, opaque tank (dimensions of 0.56 m × 0.56 m). The tank contained four grey Lego baseplates, one glued securely onto each side of the tank. Plates were placed centrally on each wall such that no positional cues could be obtained, with the tank situated below a plain white ceiling also devoid of directional cues. In each trial, two red Lego landmarks were attached to one of the baseplates; the baseplate used was rotated randomly between trials to avoid subjects utilizing geomagnetic information. Fish were released from a clear Perspex holding box (0.10 m × 0.10 m × 0.10 m) with a trap door that could be operated remotely to allow fish to exit into the test tank (Figure 1). This box could be moved about the tank and attached to the base plates also using Lego blocks such that the starting position of fish was also varied randomly between trials. Again, this prevented fish from being able to use geomagnetic cues or egocentric turning cues. A food item was attached to the uppermost surface of the Lego landmarks using Vaseline and microscope coverslips. Rewarded landmarks had a coverslip placed on top with a Tetra flake attached to it; non-rewarded landmarks had a Tetra flake attached on the upper surface but with a coverslip over the top in order that subjects could not access the food reward. Placing coverslips on both rewarded and non-rewarded landmarks ensured that subjects could not observe differences in the landmarks before a decision was made to visit one or the other.

### 2.3. Initial Training

The subjects completed a total of three x three-hour-long sessions in the testing tank in groups of eight to allow them to become familiar with the testing tank. All fish were able to swim freely during this time. Two red Lego landmarks both had food rewards attached using Vaseline, with food replenished up to three times during each three-hour-long session. Multiple feeding opportunities served to allow subjects to become accustomed to associating food rewards with the test apparatus. Fish were placed directly into the testing tank and did not experience the holding box at this point in pretraining.

Following this initial acclimatization period, fish were given additional time to become familiar with the holding box and test tank when no longer in shoals. Each fish received 15 sessions within the test tank before experimental training began. In each session, individuals were placed into the holding box for two minutes before being released into the tank and allowed to consume a food reward. After a period of 15 min, fish were removed from the training tank regardless of whether the reward had been consumed. The Lego blocks were placed centrally on the baseplates, and subjects were able to feed from both.

### 2.4. Experimental Training

**Figure 2:**
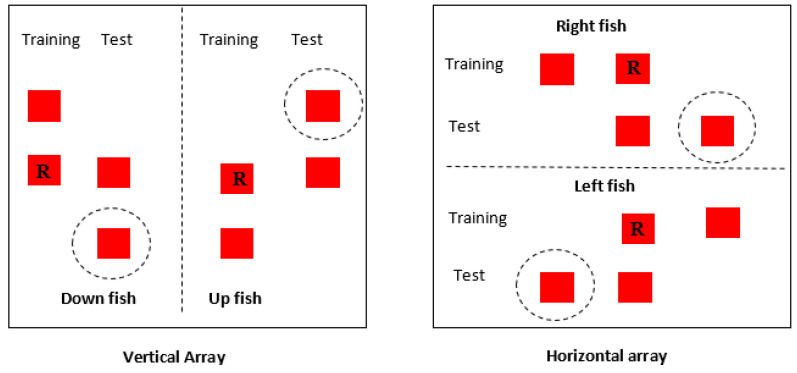
Testing and training trials for fish in the vertical and horizontal axes. The letter R indicates the rewarded stimulus. Dashed circles indicate choice when the fish does transpose and has learnt the relationship between landmarks.

Fish were placed into two experimental groups, either ‘down-right’ or ‘up-left’. Fish in the down-right condition were trained to go to the bottom landmark in the vertical array and the right-hand landmark in the horizontal array (both of these landmarks were actually in the middle of the baseplate, however, see Figure 2 for clarification). Fish in the up-left condition were trained to visit the top landmark in the vertical array and the left-hand landmark in the horizontal array. Testing in both directions helped to control for height preference as some fish were rewarded by visiting the highest landmark, while others were trained to select the lower landmark. Apart from this difference, training in the two groups was identical.

Two red Lego landmarks were presented in every trial in a predictable relationship, with trials alternating between the vertical and horizontal arrangement. Only one of the landmarks was reinforced in each trial (i.e., always the bottom/right or upper/left landmark depending on which group of fish was being trained). As noted, both landmarks had food flakes attached, but a coverslip prevented the reward from being consumed at the incorrect landmark. This ensured that olfactory signals were as similar as reasonably possible at both landmarks. As noted previously, the correct landmark also had a coverslip on top to ensure visual cues were similar; in this case, however, food was attached to the upper surface allowing subjects access to the reward. Initially, partial reinforcement was used such that fish would become accustomed to performing the task without reward; the correct landmark was therefore rewarded on 2/3 training trials. After a number of trials, however, it became clear that this was inhibiting the subjects’ learning since although behavior was unaffected in the unrewarded trial, incorrect choices were frequently made in the following rewarded trial, as subjects appeared to have unlearnt the task. Due to this, all correct choices were rewarded with food after this point.

During all experimental trials, the location of the rewarded Lego block remained constant in the axis being tested, allowing fish to learn absolute position, possibly by using hydrostatic pressure cues (in the vertical axis). Fish could therefore pay attention to absolute positional cues or could learn the relationship between the two landmarks, therefore focusing on primarily visual cues. As noted, other global landmark cues were scrambled, and subjects were prevented from using vector/egocentric methods to reach the reward by varying their release point in each training trial. Olfactory cues left by the fish/preference for any given Lego block were additionally ruled out by changing/cleaning Lego blocks between each trial.

Each fish completed four experimental trials on alternate days. All trials took place between 0800 h and 1700 h. Fish were placed into the holding box for two minutes before being released into the testing tank and given 15 min to consume the reward. Latency to first contact and which of the two landmarks was visited initially were recorded. Once the subject had visited the correct landmark and consumed the reward, it was carefully netted and returned to the holding box ready for the next trial. If after 15 min the reward had not been consumed, then the trial ended. The holding box and position of the Lego landmarks were altered between each trial. The subjects reached the criterion when they swam straight to the correct landmark in 9 out of 10 trials (defined as first contact with the rewarded rather than the non-rewarded block). The criterion had to be reached in both vertical and horizontal orientations.

### 2.5. Testing

Once subjects successfully reached both criteria, they took part in probe trials. The probe trials followed the same procedure as the training trials in that the fish were placed into the holding box for two minutes before being released into the testing tank. In the probe trials, however, neither landmark was rewarded. Two landmarks were presented in the same relationship learnt by the subjects during the training phase; however, they were shifted either horizontally or vertically. Fish that had learnt the rule ‘always visit the right-hand or bottom landmark’ were presented with landmarks shifted further to the right or downward on the baseplate. The reverse was true for fish in the up-left condition. To show transposition and reliance on visual cues therefore, fish should select the novel landmark (see Figure 2). A reliance on absolute positional cues would in contrast result in fish selecting the landmark in the same position as the previously rewarded landmark. Latency to first contact and which landmark was visited by subjects was recorded. After a decision had been made, the fish were carefully netted, and the probe trial ended.

Following a probe trial, fish underwent training trials in which they were required to select the reinforced stimulus in at least 4 out of 5 trials (in both the horizontal and vertical arrangement). Once this criterion was reached, they took part in further test trials interspersed with training trials. Each fish completed 3 test trials in each orientation (6 test trials in total).

A simplified flow diagram detailing the steps each fish underwent in training and testing is presented in the Supplementary Materials (Figure S1).

## 3. Results

### 3.1. Learning

**Figure 3:**
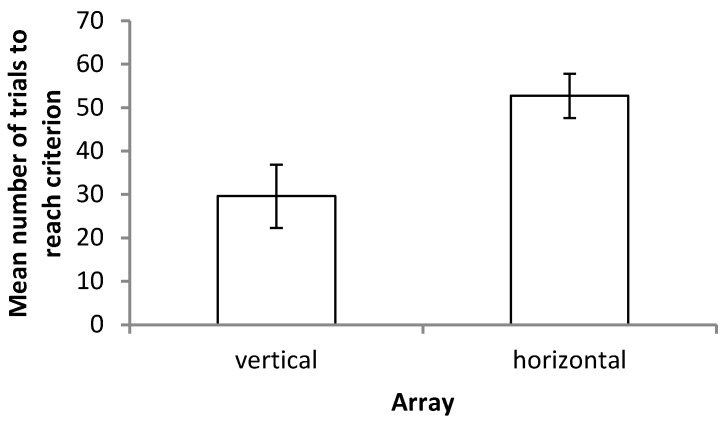
Mean number of trials required to reach the criterion (9 out of 10 correct choices) in the horizontal and vertical arrays. Error bars denote the standard error of the mean. See Figure S2 for a scatter plot showing this data.

Fourteen fish completed probe trials in at least one array; 13 reached the criterion and completed probe trials in the vertical array, and 12 reached the criterion and completed probe trials in the horizontal array. There was a large amount of individual variation in how many training trials were required to reach criterion, with some fish learning the task in as few as 9 trials and others requiring up to 80 training trials before the criterion was reached. Learning was significantly faster in the vertical axis as compared to the horizontal axis, with fish requiring an average of 29.62 ± 26.27 trials to reach the criterion compared to 52.75 ± 17.58 trials (Mann–Whitney test, *p* = 0.047), respectively (Figure 3).

**Figure 4:**
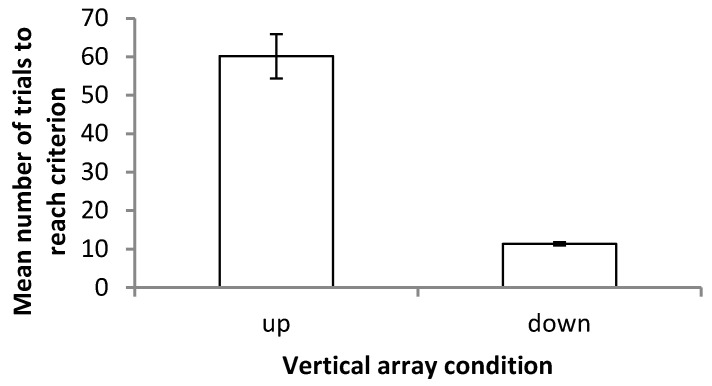
Mean number of trials required to reach the criterion (9 out of 10 correct choices) on the vertical array task for fish in ‘up’ and ‘down’ conditions. Error bars denote the standard error of the mean.

Aside from this discrepancy in axes, strong differences were also found between the number of trials required by fish in the ‘up’ and ‘down’ conditions. Fish learning to transpose downwards required significantly fewer trials to reach the criterion (mean = 10.50 ± 1.69) than fish learning to transpose upwards (mean = 60.20 ± 12.87) (Mann–Whitney test, *p* = 0.002) (Figure 4). No such difference was found between the number of trials required for ‘left’ (mean = 47.40 ± 9.07) and ‘right’ (mean = 56.57 ± 21.70) fish to reach the criterion (Mann–Whitney test, *p* = 0.530). Overall, fish in the ‘down’ condition required the least number of trials to reach the criterion and ‘up’ the most, with the horizontal array representing an intermediate between the two.

Examination of the data from the early part of the experiment in which partial reinforcement was used showed that fish did not respond differently in the reinforced and non-reinforced trials. The percentage of correct responses was not found to be significantly different between the two trial types (Mann–Whitney test, *p* = 0.568), suggesting that olfactory cues or visual differences between the two landmarks were not being utilized (see Figure S3). We can therefore be confident that fish were learning the task and not simply responding to the presence of food.

### 3.2. Landmark Preference

**Figure 5:**
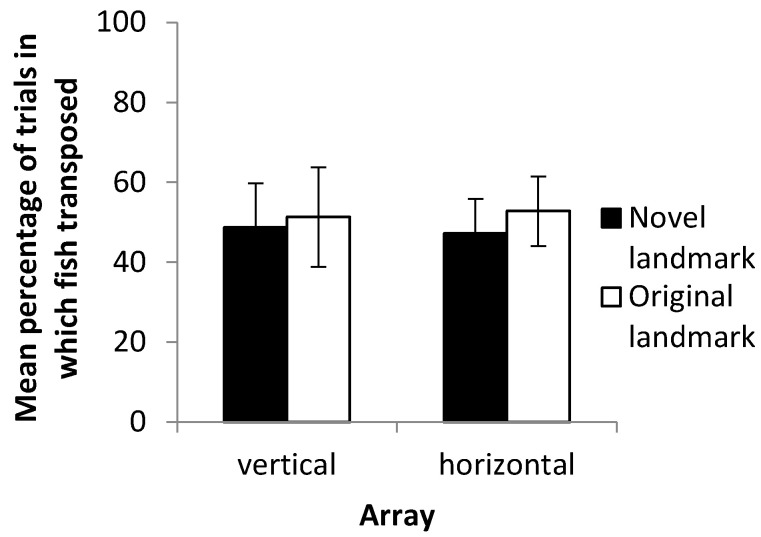
Mean percentage of trials in which fish selected the novel or previously reinforced landmark. Error bars denote the standard error of the mean.

Across all treatments, fish showed no clear preference in their response to the cue conflict experiment and appeared to select landmarks at random (Figure 5). In 39 of 77 probe trials (50.65%), fish chose the novel landmark, while in the remaining 38 trials, they chose the previously reinforced landmark. Analyzing the data for horizontal and vertical arrays separately did not alter this result, with the median percentage of transposition not being significantly different to 50% in either the horizontal (Wilcoxon signed-rank test, *p* = 0.339) or vertical (*p* = 0.839) axis. Accordingly, there was also no significant difference between the rate of transposition in the horizontal axis compared to the vertical (Mann–Whitney test, *p* = 0.80).

**Figure 6:**
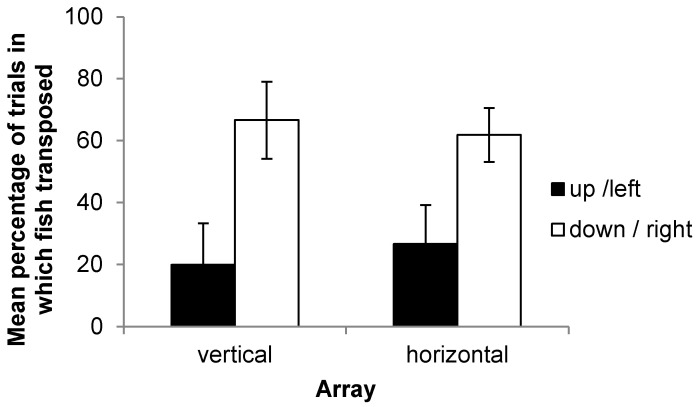
Mean percentage of trials in which fish transposed (selected the novel landmark) shown for the horizontal and vertical arrays. Error bars indicate the standard error of the mean.

Analyzing the data separately for fish in the ‘up-left’ and ‘down-right’ conditions again resulted in the conclusion that the rate of transposition was not significantly different to 50% (Wilcoxon signed-rank test: ‘up fish’, *p* = 0.125; ‘down fish’, *p* = 0.313; ‘left fish’, *p* = 0.125; ‘right fish’, *p* = 0.688). Welch’s *t*-test did, however, find that fish tested in the ‘up’ condition were significantly less likely to show transposition than those tested in the ‘down’ condition (*p* = 0.030) (Figure 6). It also appeared that fish were more likely to select a novel landmark moved to the right rather than the left, though this result was not significant (Mann–Whitney test, *p* = 0.073).

**Figure 7:**
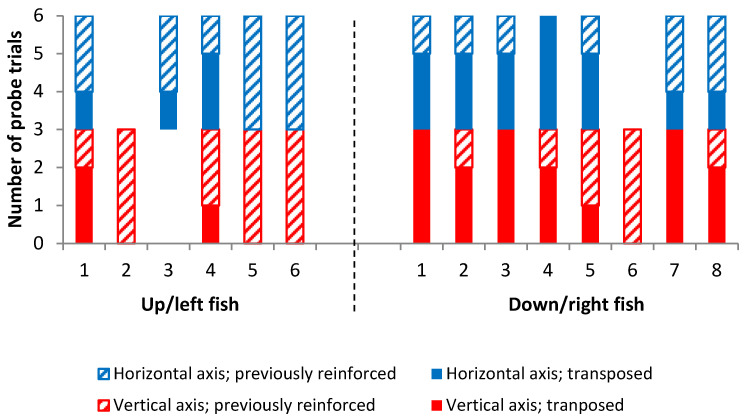
Choices of individual fish over the six probe trials. (In cases where only three trials are displayed, fish only reached the criterion and completed probe trials on one rather than both of the arrays). Note that this does not reflect the order of choices made in trials but rather the total number of each choice. The x axis shows the individual fish: 6 tested in the up/left condition and 8 in the down/right group.

In addition to there being no overall difference in preference for novel or previously reinforced landmarks, individual fish were also not consistent in their choices among the three probe trials (Figure 7). Some fish initially showed transposition in the first one or two probe trials and then switched to choosing the previously reinforced landmark. Other individuals did the reverse, initially selecting the previously reinforced landmark and then changing strategy and showing transposition. Only two fish showed a consistent response across all three probe trials on both the horizontal and vertical arrays; both of these individuals always chose to visit the previously reinforced landmark.

### 3.3. Pre-Existing Preferences

To explore the possibility that differences in the willingness to transpose down rather than up was due to a pre-existing preference, the choice made by each fish on its very first experimental trial was examined. No evidence for a pre-existing height preference was found; 13 of the 14 fish reported in this experiment visited the middle landmark first (representing the upper landmark in ‘up’ fish and the lower landmark in ‘down’ fish), and one fish visited the top landmark. There was also no evidence for a preference for either the right or left landmark in the horizontal array.

### 3.4. Latency to First Contact

**Figure 8:**
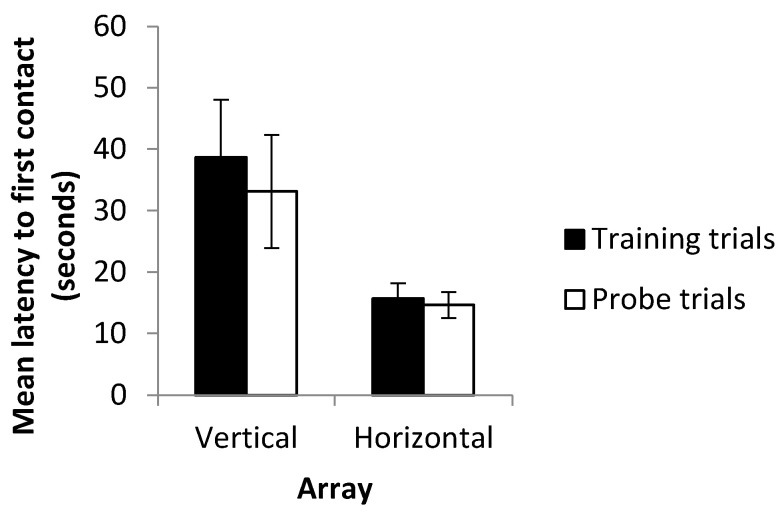
Mean Latency to first contact for probe trials and the last three training trials. Data shown separately for the vertical and horizontal arrays. Error bars denote standard error of the mean.

**Figure 9:**
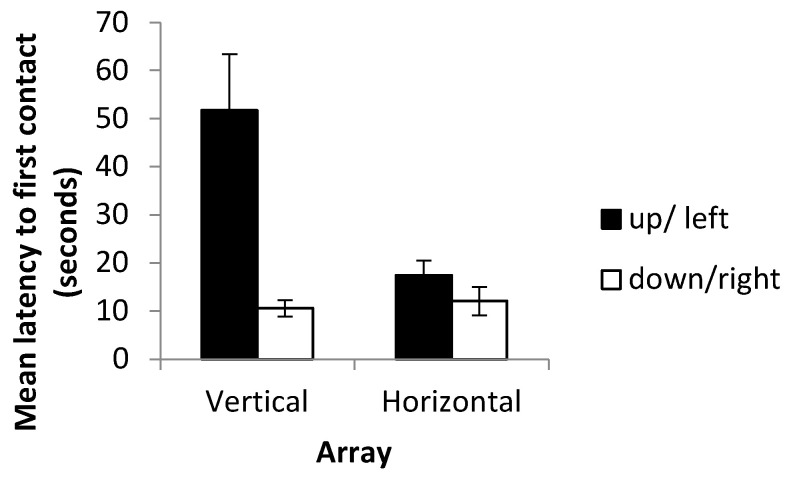
Mean latency to first contact across both the horizontal and vertical arrays showing up/left and down/right fish separately. Data include both testing and probe trials. Error bars denote the standard error of the mean.

Mean latency to first contact was not found to be significantly different between probe trials (where cues were in conflict) and the final three training trials (where cues were consistent) (*t*-test on log-transformed data, *p* = 0.799). Fish were therefore just as quick to make a decision when presented with landmarks in a novel arrangement as they had been when faced with a familiar landmark array (see Figure 8). Latency to first contact was, however, found to be significantly lower on the horizontal array (mean = 14.64 ± 9.57) than on the vertical array (mean = 35.87 ± 40.28) when using data from both the experimental and probe trials (*t*-test on log-transformed data, *p* = 0.0167). No significant difference was observed in latency to first contact between fish trained to swim right (mean = 12.09 ± 2.93) and those trained to swim left (mean = 17.40 ± 3.09) (*t*-test, *p* = 0.244). In contrast, fish were quicker to contact landmarks in the ‘down’ condition (mean = 10.59 ± 1.69) than in the ‘up’ condition (mean = 51.68 ± 11.75) (*t*-test on log-transformed data, *p* =< 0.001) (Figure 9).

## 4. Discussion

This study found no evidence that there is a significant difference in how banded tetras utilize relational/visual cues compared with positional/hydrostatic pressure cues when cues are placed into conflict. This response was consistent across both horizontal and vertical axes, with fish selecting landmarks randomly in both cases. Individual fish frequently switched their strategy when cues (and senses) were placed into conflict rather than showing individual preferences, yet did not increase their latency to contact compared to the training trials. Together, these results suggest that visual cues remain important, even when alternative cues are available; vision is no more or less important when navigating through the vertical plane compared to the horizontal.

The lack of cue preference shown by individuals ran counter to predictions and requires an examination of all of the possible outcomes of the probe trials. Four possible outcomes were identified:

Outcome One—Fish consistently select the novel landmark showing transposition and reliance on visual cues. This outcome would necessitate that they learnt something about the relative position of the two landmarks and encoded the relationship between them. Since all other cues (for example, magnetic or other cues external to the testing tank cues) were either absent or scrambled, the relationship between the landmarks is the only reliable cue; only if fish were learning the relationship between landmarks and paying attention to primarily visual cues could this result have occurred. While it cannot be excluded that the relative position of the two landmarks could be sensed using the fish’s lateral line, this is expected to be minor in comparison to the information gained from the visual stimuli.

Outcome Two—Fish continue to select the previously reinforced stimulus. They therefore learnt the absolute position in space of the landmark. In the vertical axis, this would suggest they were using hydrostatic pressure cues. In the horizontal axis, pressure cues are unavailable, suggesting that absolute position may be encoded with reference to the sides of the tank, possibly using their lateral line. This would require that fish had at least some concept of either distance from the edge of the tank or, at the very least, of being near to or far from the edge of the tank.

Outcome Three—Some fish select the novel stimulus, while others select the previously reinforced stimulus. In this outcome, fish were presumably able to learn both the relative and absolute position of landmarks. The differences in the responses of subjects would then be linked to individual preferences for which cues were perceived to be more reliable/salient. Such individual preferences have been seen in previous studies on fish [25].

Outcome Four—Fish select landmarks by chance, sometimes visiting the previously reinforced landmark, sometimes visiting the novel landmark. This could suggest that fish were unable to learn about relative and absolute positions. The fact that fish reached the criterion of nine out of ten correct responses before undergoing probe trials, however, shows that this is not the case. It is likely therefore that information about both absolute and relative position (and therefore multiple sensory systems) is used ordinarily by fish; when these are put into conflict during probe trials, fish may be confused and select landmarks at random. This would suggest that neither visual cues nor hydrostatic pressure cues are considered more reliable or important than the other.

Outcome Four best summarizes the results of the experiment. In all but two of the fish tested in both arrays, there was variation in landmark choice across trials, with no consistent strategy of always transposing or always choosing the previously reinforced landmark. In the two individuals that did show consistent strategies, the absolute position of the landmark was preferred, with fish always selecting the previously reinforced stimulus. As noted, it is not the case that fish were unable to learn the task as they were able to consistently select the correct landmark in reaching the criterion. It is also thought to be unlikely that fish treated the training and testing phases as unrelated due to the small shift in landmark position; during training, both the starting box and the landmarks were rotated around the four walls at random, meaning that any two training trials were more different to each other than the smaller difference caused by the slight shift in landmark positions for the probe trials. It would appear therefore that both sets of cues (and multiple senses) are required for fish to make an informed decision. These results contrast those of Holbrook and de Perera [22] in which banded tetras disregarded landmark cues once they became unreliable and preferentially used hydrostatic pressure to navigate. Similar results have, however, been obtained in a previous study; in tests with goldfish, Vargas et al. [26] found that when previously reliable cues were put into conflict, fish began to make choices at random and did not appear to have a preference for using one cue over another.

Although no quantifiable data were collected and the observation is anecdotal in nature, it was noted that, especially in the horizontal probe trials, fish occasionally appeared to exit the holding box and began to swim toward the landmarks, pausing briefly before finally approaching a landmark. This would appear to be a reasonable behavior if subjects were indeed using multiple (now conflicting) cues in order to make a decision. Despite this, latency to first contact was not found to increase during the probe trials when compared to the training trials. Sutherland et al. [19], however, reported that while trajectory length increased in blind cavefish when cues were placed into conflict, swimming speed increased such that latency to contact remained unchanged. Since paths swum by the fish were not recorded, it is not possible to determine whether such differences in swimming speed or in tortuosity existed in this experiment. Differences between latency to first contact in the vertical and horizontal axes were, however, clearer, with fish approaching the landmarks more rapidly in horizontal trials than in vertical trials under both the training and probe conditions. Approaching landmarks more slowly in the vertical trials contrasts with the decreased number of trials required to reach the criterion in this array. A possible explanation may be that having cues relating to multiple senses present (in the form of visual landmarks and hydrostatic pressure gradients) enables more rapid learning of the vertical task, therefore reducing the number of trials required to reach the criterion. Multiple streams of information may, nevertheless, come with an increased information processing load leading to an increased latency to contact in the vertical array. Further examination of the data, however, revealed strong differences between the ‘up’ and ‘down’ conditions, with latency to contact much higher in fish trained to transpose upwards. Alternatively, increased latency in the vertical array may simply reflect an unwillingness to transpose upwards (as detailed below) rather than be indicative of reductions in speed resulting from increased information processing. These results contrast with those of previous studies in which learning rate did not differ between the horizontal and vertical axes, and with or without multiple cues available [22,24]. They also strongly contrast with previous experiments showing that latency to first contact was more rapid when multiple cues were available [22], possibly suggesting the second explanation to be more likely.

It is notable that many of the fish tested in the horizontal axis chose the previously reinforced stimulus in at least some of the probe trials. This was despite the fact that there was no hydrostatic pressure gradient or any other positional cues (either local or global) available to aid this decision. As noted, the only cue available to fish to allow them to navigate toward the previously reinforced stimulus was the distance from the sides of the tank. It remains possible that subjects were able to gain information about their position with respect to the side walls via their lateral line or other mechanosensory senses. Although it has not been demonstrated that fish can measure distance in a metric sense, experiments by Warburton [8] showed that goldfish could use landmarks that indirectly signaled a food reward buried in gravel. To locate the reward, they needed to explore an area further away from, rather than closer to, the landmark. This could be similar to the strategy used by fish in this experiment when choosing the previously reinforced stimulus if they were swimming ‘far away from’, rather than ‘closer to’, the edge of the Lego baseplate or sides of the tank. In practice, however, all landmarks were placed broadly centered on the walls such that a small shift would still leave them far from the edge of the tank. Nevertheless, it is an interesting result as it shows that the mixed result on the vertical array was not simply due to the conflict between hydrostatic pressure and visual cues.

Examining the choices of subjects in their first experimental trial made it clear that fish did not have a pre-existing height preference that could explain the differences observed between the chance of transposition in ‘up’ fish compared to ‘down’ fish. Fish did not universally prefer the lower of the landmarks prior to training. The result that transposition was more likely to occur downwards therefore appears striking. However, in order to transpose upwards, a fish would be required to swim relatively close to the water surface. Observations in this experiment brought to light that subjects were somewhat reluctant to do so and most often spent their time in the mid water or lower section of the tank. Similarly, Holbrook and de Perera [24] report that fish were unwilling to swim upwards at a steep angle in experiments using a rotating Y maze. They suggest that this may be an anti-predator response that is still displayed under lab conditions. An unwillingness to transpose upwards may therefore be a reflection of this rather than any difficulty in learning to transpose upwards per se. Accordingly, this may also explain the increased latency to first contact on the vertical as opposed to horizontal axis and the differences in learning rate between the up and down conditions. It is an unfortunate limitation of the study, and it would be desirable to conduct experiments in a larger testing tank in which swimming upwards does not require fish to swim close to the surface of the water.

This study has demonstrated that fish do not appear to show significantly different responses or use different cues to solve a transposition task within the horizontal and vertical axes. In contrast to our predictions, no evidence was found for a greater reliance on the absolute cues provided by a hydrostatic pressure gradient in the vertical axis. Visually based cues remain important to fish even when potentially more stable cues are available. In both horizontal and vertical tasks, fish selected landmarks at random once relational and absolute cues were placed into conflict. It therefore appears that under normal circumstances, both sets of cues and multiple sensory systems are used to gain information about their environment. Our study therefore provides further evidence that, rather than being driven by a single sensory system, much of animal behavior is mediated by a synergy of sensory information.

## Data Availability

Data available on request from the corresponding author.

## Supplementary Materials

The following supporting information can be downloaded at: https://www.mdpi.com/article/10.3390/vision7020044/s1. Figure S1: Diagram showing the steps each fish went through in training and testing. Each fish completed three probe trials in the vertical array and three in the horizontal array. Figure S2: Scatterplot showing the number of trials each fish took to reach criterion on the horizontal and vertical arrays. Figure S3: Mean percentage of trials in which fish selected the correct landmark when a food reward was either present (rewarded trials) or absent (non-rewarded trials). N = 209 in rewarded trials and 70 in non-rewarded trials. Error bars denote standard error of the mean.
