# Supplementary material for: Vision in the Vertical Axis: How Important Are Visual Cues in Foraging and Navigation?
Source: Vision (Basel). 2023 Jun 6;7(2):44. doi: 10.3390/vision7020044 (PMC10304561; doi:10.3390/vision7020044)
Supplement: Supplementary file 1 [file vision-07-00044-s001.zip › vision-2058183-supplementary.pdf]

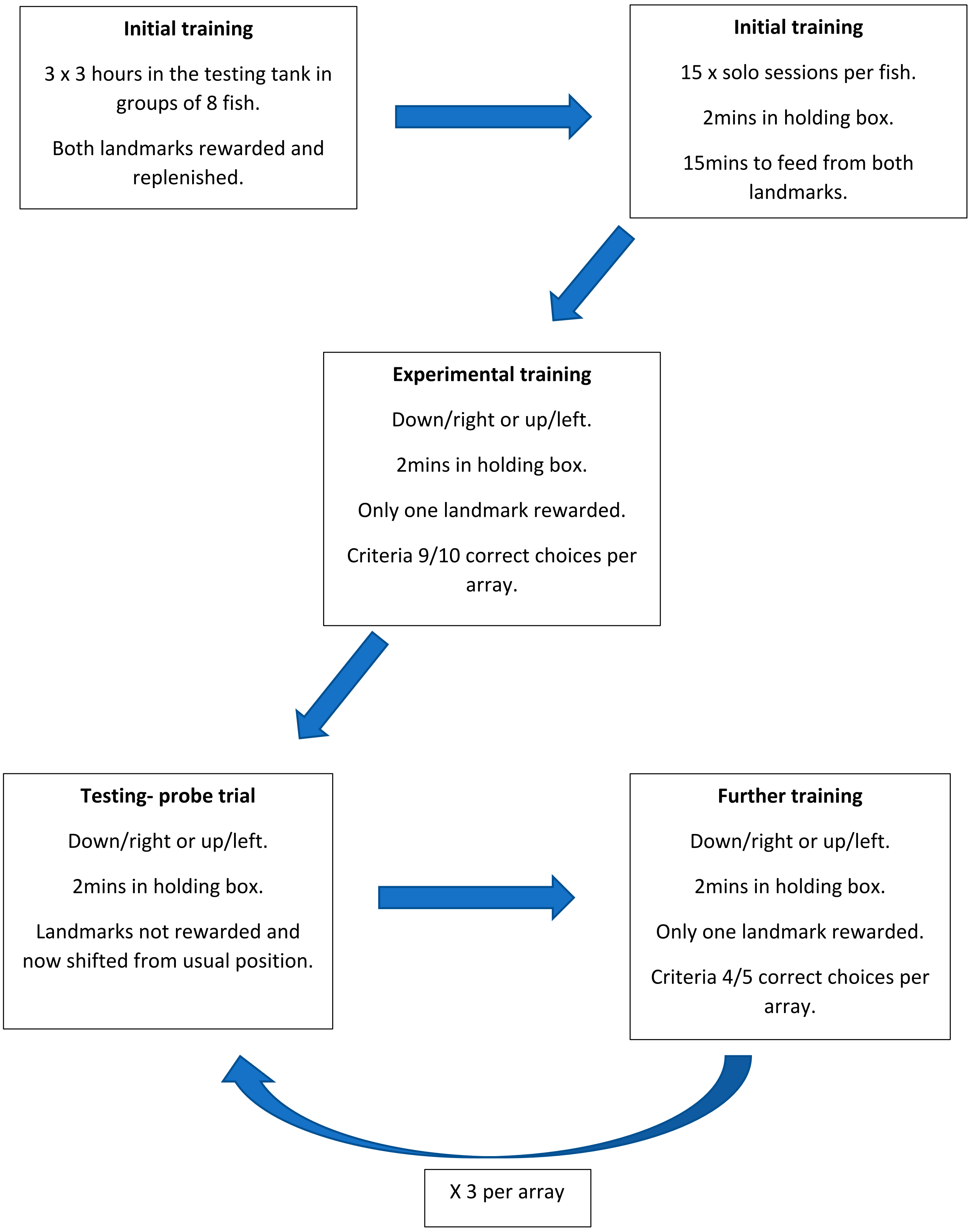

Figure S1; Diagram showing the steps each fish went through in training and testing. Each fish completed three probe trials in the vertical array and three in the horizontal array.

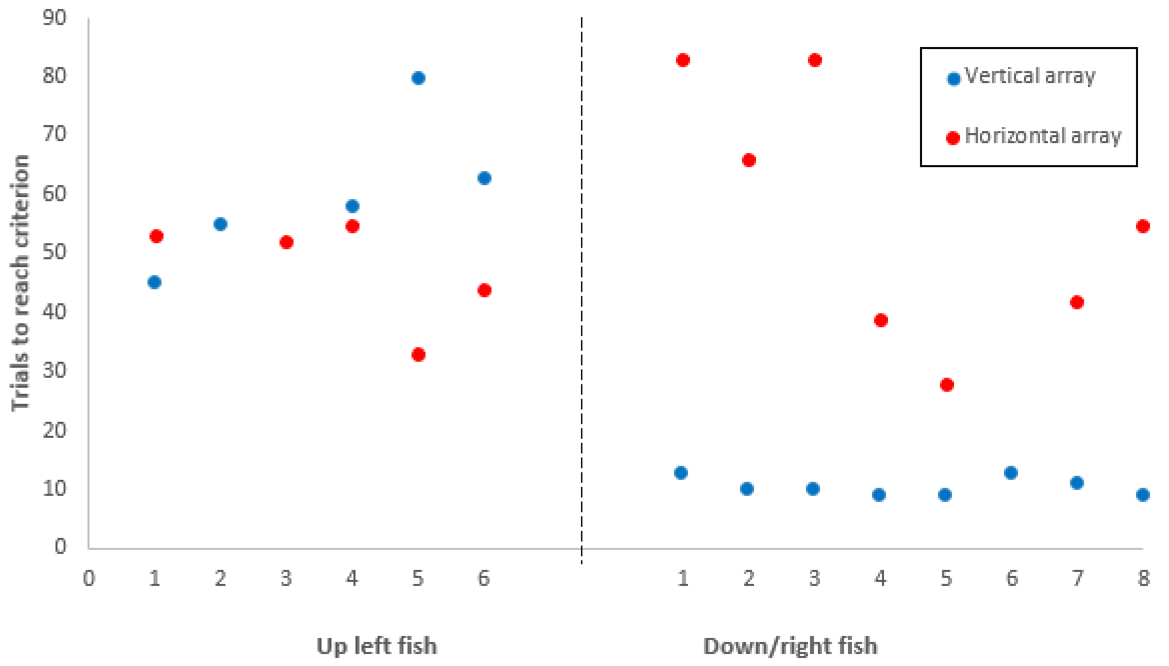

Figure S2; Scatterplot showing the number of trials each fish took to reach criterion on the horizontal and vertical arrays.

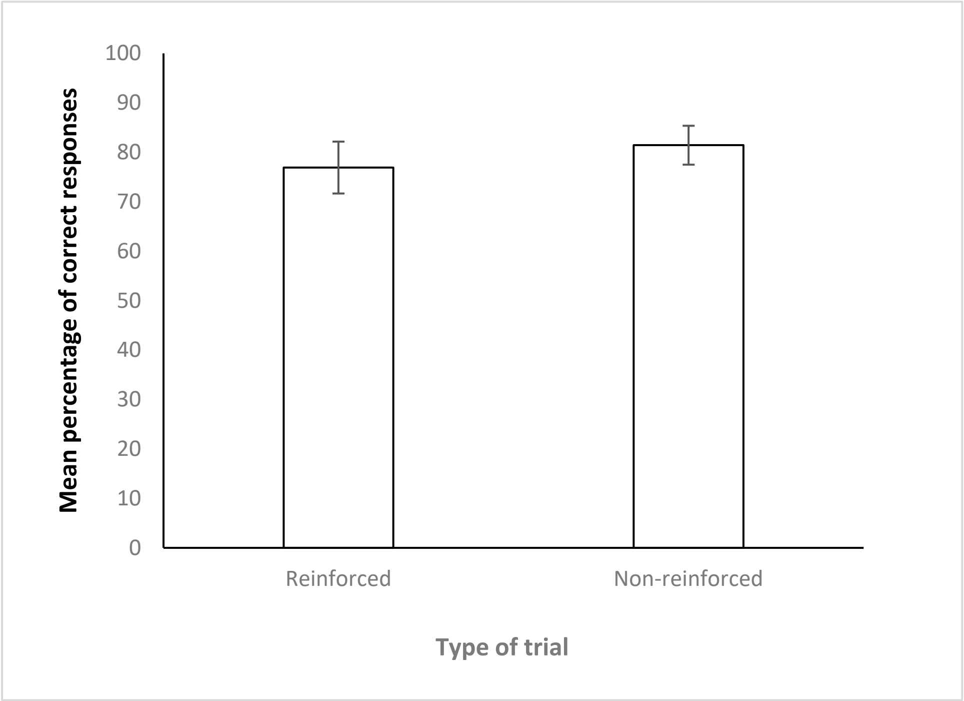

Figure S3; Mean percentage of trials in which fish selected the correct landmark when a food reward was either present (rewarded trials) or absent (non-rewarded trials). N= 209 in rewarded trials and 70 in non-rewarded trials. Error bars denote standard error of the mean.
